# Correlation of Epididymal Protease Inhibitor and Fibronectin in Human Semen

**DOI:** 10.1371/journal.pone.0082600

**Published:** 2013-12-16

**Authors:** Xiangxiang Zhang, Jianzheng Fang, Bin Xu, Shengli Zhang, Shifeng Su, Zhen Song, Yunfei Deng, Hainan Wang, Dan Zhao, Xiaobing Niu, Zengjun Wang

**Affiliations:** 1 State Key Laboratory of Reproductive Medicine, Department of Urology, First Affiliated Hospital of Nanjing Medical University, Nanjing, China; 2 Department of Urology, Affiliated Zhongda Hospital of Southeast University, Dingjiaqiao, Nanjing, China; 3 Department of Urology, Jiangsu Provice Official Hospital, Nanjing, China; Van Andel Institute, United States of America

## Abstract

**Objective:**

Epididymal protease inhibitor (Eppin) was located on the surface of spermatozoa and modulates the liquefaction of human semen. Here, we identify the correlative protein partner of Eppin to explore the molecular mechanism of liquefaction of human semen.

**Methods:**

(1) Human seminal vesicle proteins were transferred on the membrane by Western blotting and separated by 2-D electrophoresis and incubated in recombinant Eppin. The correlative protein was identified by Mass Spectrometry (MS) (2). Western blotting was used to determine the relation of rEppin and rFibronectin(Fn); (3) Co-localization in spermatozoa were detected using immunofluorescence; (4) Correalation of Eppin and Fn was proved by co-immunoprecipitation.

**Results:**

Fn was identified as the binding partner of recombinant Eppin by MS. Recombinant of Eppin was made and demonstrated that the Eppin fragment binds the fn 607-1265 fragment. The Eppin-Fn complex presents on the sperm tail and particularly in the midpiece region of human ejaculated spermatozoa. Immunoprecipitation indicated that Eppin in the spermatozoa lysates was complexed with Fn.

**Conclusions:**

Our study demonstrates that Eppin and Fn bind to each other in human semen and on human ejaculated spermatozoa. Eppin-Fn complex may involve in semen coagulation, liquefaction and the survival and preparation of spermatozoa for fertility in the female reproductive tract.

## Introduction

 Eppin (SPINLW1; GeneID, 57119), a single-copy gene, is located in a telomeric cluster on human chromosome 20q12-q13. Structural analysis of this gene suggests that it contains Kunitz-type and WAP-type sequence, which are connected by four-disulfide bonds and belongs to the whey acidic protein (WAP)-type protease inhibitor gene family[1]. In human, Eppin gene, encodes cysteine-rich proteins, is tissue specifically expressed only in testis and epididymis [1-3]. It has been found that Eppin is highly expressed in human spermatozoa and seminal plasma [4]. On the surface of ejaculated spermatozoa, Eppin becomes in a complex of proteins containing lactotransferrin clusterin and Sg[5]. Eppin has the potential of being an essential molecule in the pre-fertilization preparation of human spermatozoa in the male reproductive tract and in the ejaculate coagulum. Antibodies directed at Eppin have been demonstrated to be an effective and reversible male immunocontraceptive in primates [6]. Therefore, it is important to characterize its function.

 Fibronectin is a dimeric filament-forming 440 kDa glycoprotein consisting of two similar 200-250 kDa subunits connected by disulfide bridges[7-9]. It is present in a soluble form in plasma and other body fluids, and in an insoluble (cellular/fibrillar) form in the fibrin clot, the loose connective tissue, and basement membranes[8-10]. Fn plays a role in diverse processes, ranging from immune adherence of microbes to connective tissue remodeling and embryogenesis [8,11-13].During sperm–egg fusion, the RGD sequence of Fn binds to the RGD receptors to facilitate sperms capacitation[14,15]. Immunofluorescence studies indicate that Fn is highly expressed on the surface of ejaculated spermatozoa and can be a marker for human sperm maturation[16]. While the expression of Fn on the surface of capacitated human spermatozoa was detected a significant increase, compared to fresh sperm, which plays a vital role in sperm capacitation [7].

Proteomic analysis of human seminal fluid has led to more detailed analysis and has indicated a large number of extracellular proteins, proteases and other proteins secreted by testes, prostate and other male accessory glands[17]. Proteins from seminal vesicles such as Semenogelin (Sg) and Fibronectin (Fn) play an important role in semen coagulation. After ejaculation, Sg and Fn aggregate to form a gelatinous mass that is liquefied within 5-20 min which releases the trapped spermatozoa. Liquefaction occurs through cleavage of Sg by PSA (prostate-specific antigen)[3,18-20]. During the process of liquefaction, PSA hydrolyzes Sg, which allows the spermatozoa to be motile and capacitated [3]. Previous studies have found that the C-terminal of Eppin (amino acids 75-133) in semen binds a fragment of Sg (amino acids 164-283) that was a specific inhibitor of PSA activity, which suggested that Eppin, Sg and PSA were involved in human semen liquefaction[4,21].

 However, the function of seminal proteins at the molecular level is still insufficiently explored. Therefore, the aim of this work was to study the function of Eppin and identify its partner proteins in human seminal fluid, which can bind to Eppin and involve in human semen coagulation and liquefaction.

## Materials and Methods

Approval for this study was granted by the ethics committee of the First Affiliated Hospital of Nanjing Medical University in Nanjing, China prior to sample collection. All participants signed consent forms. The Eppin plasmid (pET-28a) was donated by Prof. Xinru Wang. Chemicals and reagents used in the present study were obtained from Sigma-Aldrich (St. Louis, MO, USA). Immobilon-P and -N transfer membranes were purchased from Millipore (Bedford, MA, USA). Fn 607-1265(amino acids 607-1265) was purchased from Sino Biological Inc.

### Seminal Vesicle Fluid Collection

Specimen was collected from a fertile patient who received radical prostatectomy at Department of Urology, the First Affiliated Hospital of Nanjing Medical University in Nanjing, China. Seminal vesicle fluid was collected by direct needle aspiration of the ligated gland. The fluid was diluted in an equal volume of PBS (pH 7.4) and kept at -70°C until used. 

### Sperm preparation

Consent was obtained from all participants (Clinical Reproductive Center, First Affiliated Hospital of Nanjing Medical University). The semen samples were obtained by masturbation after at least 3 days of abstinence from 10 healthy male volunteers of proven fertility and with normal semen quality, as assessed by World Health Organization criteria (2010). The semen samples had the following characteristics: sperm concentration (n×10^6^/ml) ≥15; Volume (ml) ≥1.5; motility (PR+NP %) ≥40; Leukocytes (n×10^6^/ml) <1; pH 7.2-7.8. The samples were ejaculated into sterile containers and allowed to liquefy for at least 30 min before being processed by centrifugation in a 60% Percoll gradient (GE Healthcare, Waukesha, WI, USA) to remove round cells.

### Recombinant Protein Production

An Eppin cDNA (nucleotides 70–423) was generated by PCR using the eppin-1/Bluescript clone as template. This PCR was performed and cloned into pET-100D/TOPO (Invitrogen). To produce N- and C-terminal fragments of Eppin, PCR products were generated with appropriate restriction enzyme sites added to the primers to allow directional cloning into pET-100D/TOPO (Invitrogen). The N-terminal Eppin cDNA construct (nucleotides 73–288) expresses amino acids 18–76 (Eppin18–76) and the C-terminal Eppin construct (nucleotides 283–423) expresses amino acids 75- 133 (Eppin75–133).

The Eppin plasmid was then transformed into the Escherichia coli BL21 (DE3) and was cultured overnight in Luria Bertani (LB) medium supplemented with kanamycin. The successfully transformed E. coli was picked up and inoculated to kanamycin-containing LB medium and grown at 37°C under continuous shaking until the absorbance at 600 nm reached 0.6-1.0 Fusion protein expression was induced with 1 mmol/L isopropy-b-D-thiogalactoside (IPTG). The recombinant proteins were purified from a bacterial lysate with 50% Ni– NTA resin and then stored at -80°C until use. 

### SDS-PAGE in two dimensions and Western blot analysis

The fluid of human seminal vesicle was mixed with non-reducing buffer and boiled 3 minutes before was analyzed by 12.5% non-reduced gradient SDS gel. Protein staining band was excised, followed by incubation with 1%DTT, to assume the reduced condition. The gel lane was flat on the top of 12.5% SDS gels with reducing buffer, which separated the proteins again. SDS gels was stained overnight with 0.01% Bio-Rad R-250 Coomassie in 10% acetic acid and then soaked in destaining solution for 1-2 hours. SDS gel was transferred to Immobilon-P (Millipore) and either stained for protein with amido black (Stain the membrane for 5 minites with Amido Black, rinse the stained membrane for 30 to 45 sec with TBST three times)orblocked with TBS-T (20nM Tris–HCl, 137 mM NaCl, and 0.1% Tween-20, PH 7.6) containing 5% skim milk, followed by incubation with 6-His-Eppin protein at 4°C overnight in order to find the protein which can bind to 6-His-Eppin protein. The next day, membranes were incubated with anti-His (1:1000, Beyotime) at 4°C overnight. After that, membranes were incubated with goat anti-mouse HRP-conjugated secondary antibody (1:2000, Beyotime) for 1 h at 37°C and visualized using enhanced chemiluminescence (Millipore). The negative controls were incubated without 6-His-Eppin protein.

For far-Western blots, 20mg of rEppin or rFn (Sino Biological Inc) were immobilized on Immobilon-P, blocked as above, incubated 1-2 h or overnight (4°C) in either rEppin or rFn and without rEppin or rFn (control), The next day, membranes were incubated with Fn mAb (1:500, Santa Cruz Biotechnology) or rabbit Eppin antibody (1:1000, Santa Cruz Biotechnology) at 4°C overnight. After that, membranes were incubated with goat anti-mouse or goat anti-rabbit HRP-conjugated secondary antibody (1:1000, Beyotime) for 1 h at 37°C and visualized using enhanced chemiluminescence (Millipore).

### Mass Spectrometry Identification of Eppin Binding Partners

SDS gels (two-dimensional) were stained overnight with 0.01% Bio-Rad R-250 Coomassie in 10% acetic acid. The protein spot which can bind to rEppin was excised, The gel fragments were thoroughly washed with 25 mmol/L NH_4_HCO_3_, 50% acetonitrile and 100% acetonitrile and dried in a Speedvac. The dried fragments were the rehydrated in 2-3μL of a 10-ng /μL solution of trypsin ( Promega, Madison, WI, USA) in 25 mmol /L NH_4_HCO_3_at 4°C for 30 min. The excess liquid was discarded, and the gel plugs were incubated at 37°C for 12 h to allow for digestion of the gel. Trifluoroacetic acid (TFA) was added to a final concentration of 0.1% to stop the digestion. The digests were immediately analyzed by MALDI/TOF (matrix-assisted laser desorption ionization/time of flight). MALDI/TOF and MALDI/TOF/TOF were performed on an AB 4700 Voyager-Proteomics Discovery System (Applied Biosystems, Foster City, CA). The resulting peptide peaks were searched against the MSDB and NCBI databases using the MASCOT search engine. Mass spectrometry (MS) identification was done in the Nanjing Medical University.

### Immunofluorescence

For immunofluorescence analysis, samples from normal subjects were prepared as described above and air-dried onto poly-lysine-coated slides. Then fixed with 4% paraformaldehyde in phosphate-buffered saline (PBS) for 40 min and permeabilized with 0.2% Triton X-100 in PBS for 20 min at 37°C. After three 5-min washes with PBS, the slides were blocked with goat serum (Beijing Zhong Shan Biotechnology Co) diluted in PBS for 1h and then incubated with mouse Fn antibody (1:250, Santa Cruz Biotechnology), rabbit Eppin antibody (1:500, Santa Cruz Biotechnology) overnight. After incubation with fluorescein isothiocyanate (FITC) or Cy3 (cyanine-3) conjugated goat anti-rabbit IgG or anti-mouse IgG (1:100; Beyotime.) for 1 h, the slides were washed in PBS and covered with a coverslip. The slides were viewed using an Axioskop 2 Plus microscope (Carl Zeiss, Oberkochen, Germany) with a × 100 oil objective, and the images were captured with a CCD camera (Carl Zeiss) using AxioVersion 4.5 software (Carl Zeiss). The negative controls were incubated with isotype mouse antibody and rabbit antibody (Santa Cruz Biotechnology) instead of the primary antibody.

### Co-immunoprecipitation (Co-IP)

Spermatozoa from at least three healthy volunteers were lysed, as described above, using RIPA buffer (1% [v/v] NP-40, 0.1% [w/v] SDS, 0.5% [w/v] sodium deoxycholate, 0.05 mol L-1 Tris, 0.15 mol L-1 NaCl, 1 mmol L-1 NaF and 1 mmol L-1 Na_2_VO_3_·12H_2_O) supplemented with 1% (v/v) protease inhibitor cocktail (Pierce) instead of lysis buffer. An equal amount of protein (500 µg) was incubated with 10µl mouse Fn antibody (Santa Cruz Biotechnology) for 1.5h at 4°C followed by addition of protein A/G Plus-agarose beads (Santa Cruz Biotechnology) and incubation for an additional 1.5h at the same conditions. The protein/Ab/beads mix was then washed three times with RIPA buffer by centrifugation at 2500× g for 5 min at 4°C and boiled in 40 μL of SDS buffer for 5 min. The samples were then separated on 12.5% SDS-polyacrylamide gel electrophoresis gels and subjected to western blot analysis, using rabbit Eppin antibody (1:1000, Santa Cruz Biotechnology) and goat anti-rabbit HRP-conjugated secondary antibody (1:1000, Beyotime). The negative controls were incubated with normal rabbit IgG as a negative control.

## Results

### SDS-PAGE in two dimensions and Western blotting analysis of seminal vesicle fluid

**Figure 1:**
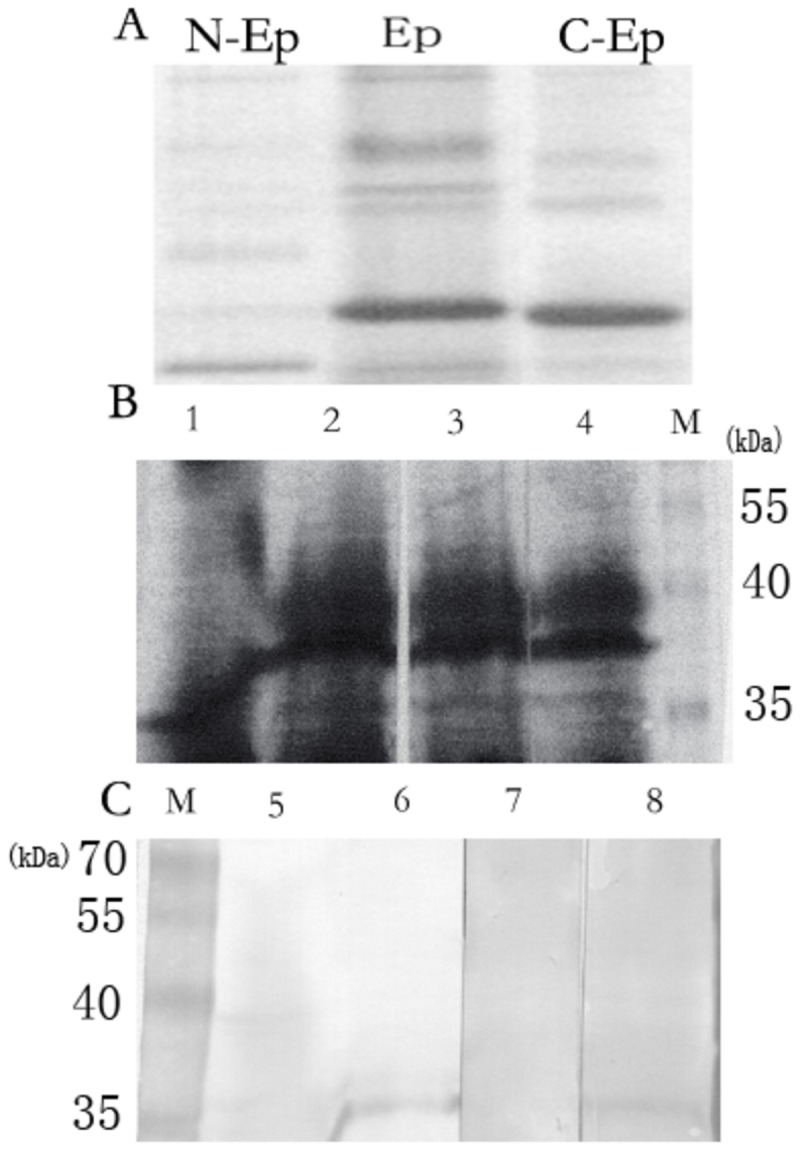
Far-western immunoblot analysis demonstrating that only the N-terminal of recombinant epididymal protease inhibitor (N-rEppin) and rEppin can binds to reducing seminal plasma proteins. A The monomer and multimers of rEppin, N-rEppin, C-rEppin in Western-blotting; N-Ep: N-rEppin (Eppin_18-76_Mr 10000); Ep: rEppin (Mr 18000); C-Ep: C-rEppin (Eppin_75-133_Mr 12000); B: Amido Black Staining of seminal plasma proteins(Stain the membrane for 5 minites with Amido Black, rinse the stained membrane for 30 to 45 sec with TBST three times),; lane 1: Non-reducing seminal plasma proteins, lane 2-4: Reducing seminal plasma proteins; M: marker; C: M: marker; lane 5-6: lane 1-2 (B) incubated with rEppin and probed with anti-his tag; lane 7: lane3 (B) incubated with C-rEppin and probed with anti-his tag; lane 8: lane 4(B) incubated with N-rEppin and probed with anti-his tag.

 rEppin and its C-terminal and N-terminal were transferred onto Immobilon-P Polyvinylidene Difuoride(PVDF) membrane by Western blot (Figure 1 A). Far-western immunoblot analysis demonstrates that the N-terminal of rEppin and full-length rEppin can bind to the specific protein fragment on the membrane (Figure 1 B, C). In reducing condition, Eppin complex or hydrolytic fragment could still be detected (Figure 1C lane 5).

**Figure 2:**
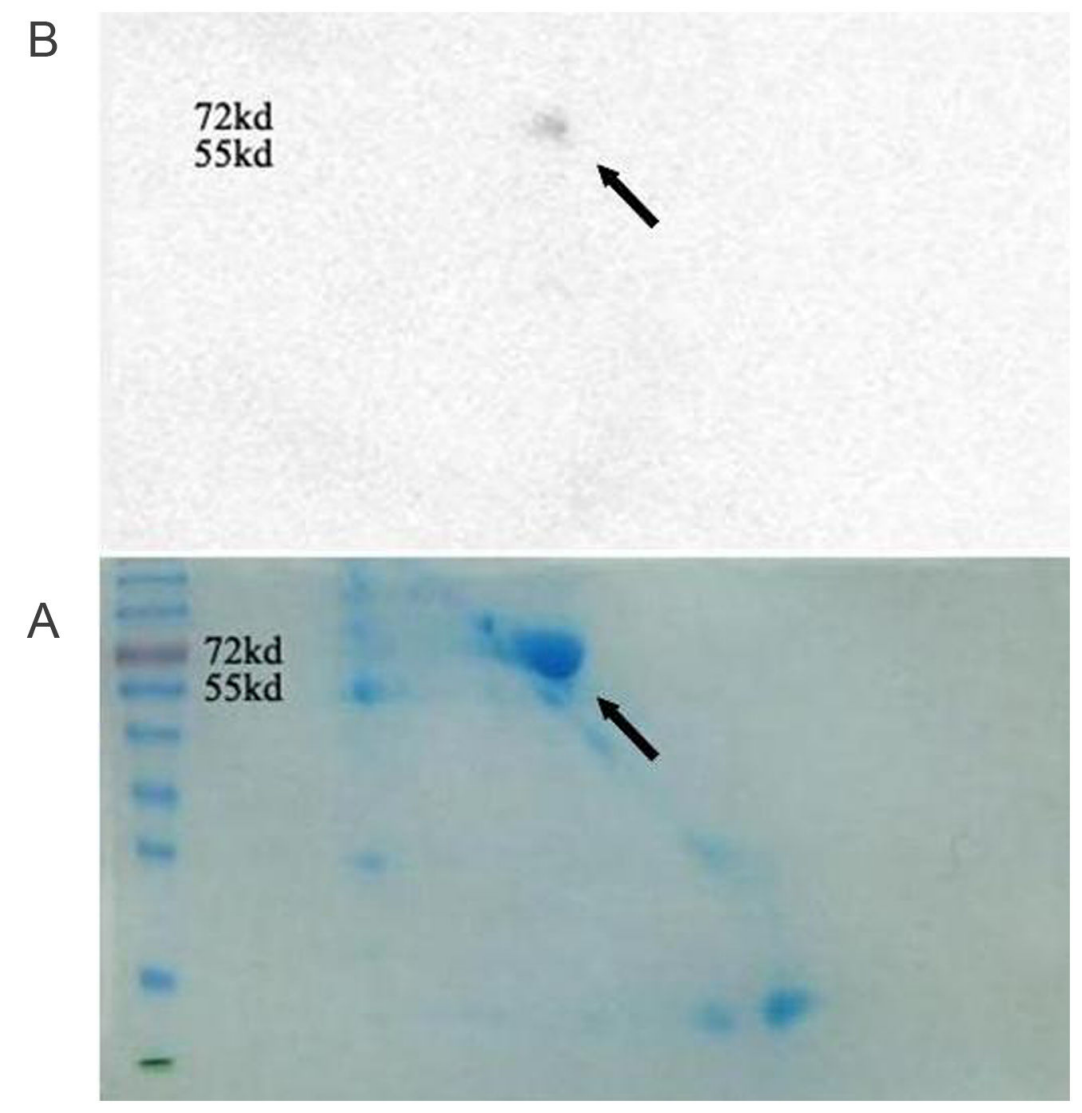
SDS-PAGE in two dimensions and Western blot analysis of seminal vesicle fluid shows a molecular weight 55-72KDa spot (arrowhead) binded to Eppin. A. Seminal vesicle fluid was separated by 2-D electrophoresis\ and was stained overnight with 0.01% Bio-Rad R-250 Coomassie in 10% acetic acid. B. Far Western blot: Incubate the membrane with 6-His-Eppin protein and probed with anti-His. We can see the positive spot molecular weight 55-72KDa on the blot (arrowhead).

SDS-PAGE in two dimensions and Western blotting analyses of seminal vesicle fluid was performed to investigate the presence of a receptor of Eppin .The proteins in seminal vesicle fluid were isolated to some spots by SDS-PAGE in two dimensions (Figure 2A). Western blotting after 2-D electrophoresi showed that the 2-D gel contained one strongly staining protein spot at approximately 72 and 55 kDa, which can bind to Eppin (Fig. 2A.B).

### Mass Spectrometry Identification of0 a receptor of Eppin

**Figure 3:**
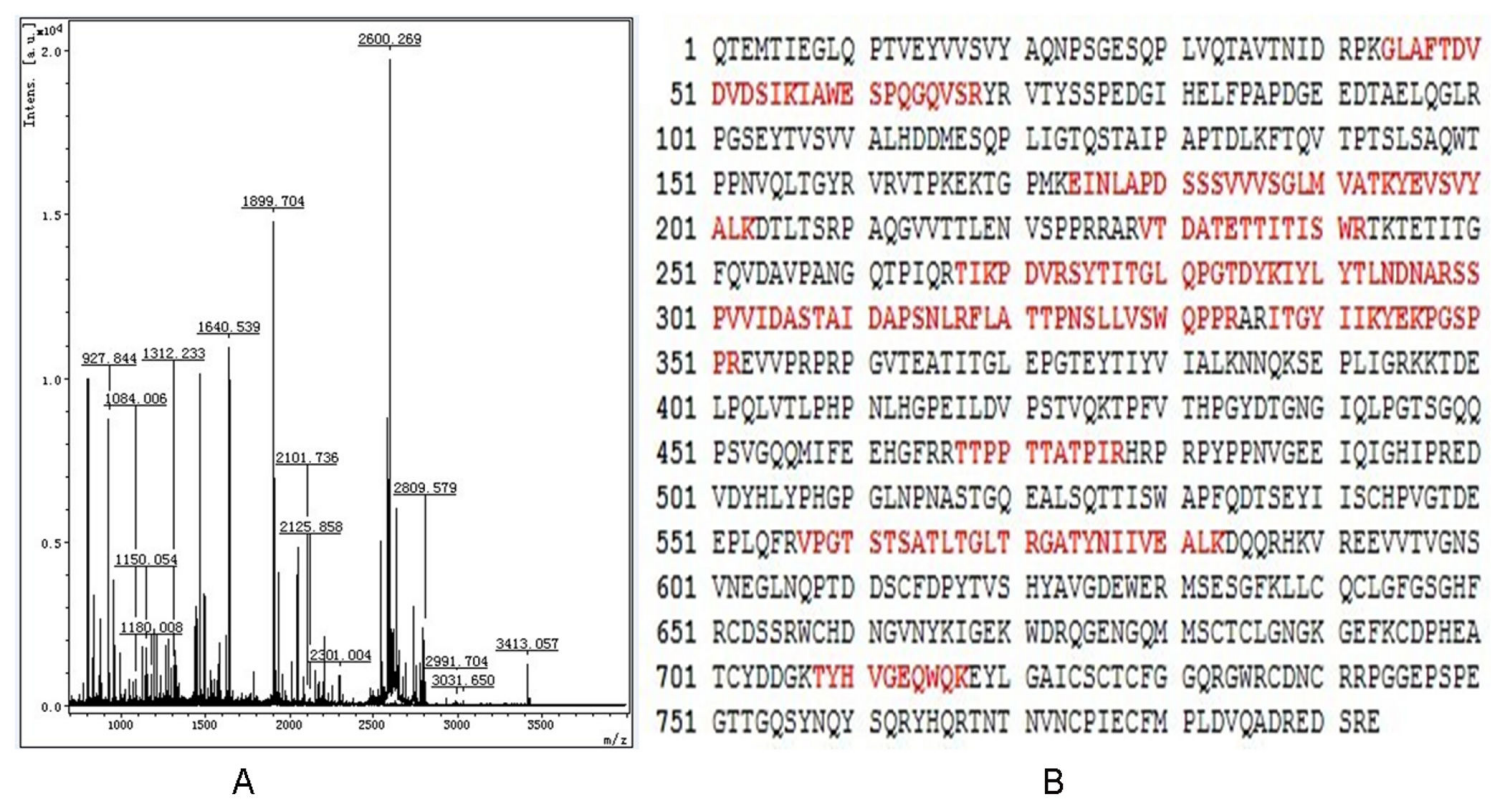
Mass spectrometric identification of Fn. A. Seminal vesicle fluid was separated by SDS gels (two-dimensional), stained Coomassie blue stained, and the protein spot trypsin digested for identification. B. Matched peptides were shown in Bold Red.

The protein spot which can bind to Eppin obtained by 2-DE was excised for MS/MS analysis. For the database search, each acquired mass spectrum (m/z, range 700–4 000) was processed using FlexAnalysis v2.4 software (Bruker Daltonics) using the following parameters: peak detection algorithm: Sort Neaten Assign and Place; S/N threshold: 3; quality factor threshold: 50. The result of the MS/MS analysis of spot was shown in Figure 3A. The peptide masses obtained were used to search the Swiss-Prot database (http://www.matrixscience.com/) (12273660 sequences, 4194265988 residues, for Human 232145 sequences) using Mascot (v2.1.03; Matrix Science, Boston, MA, USA) in the automated mode. The tryptic peptides were identified as human Fn and matched peptides were shown in Bold Red and sequence coverage: 25% (Figure 3B). Fibronectin was the partner of Eppin and we need to further verify the interaction between them.

### Western blotting analyses of the interaction between rEppin and rFn

**Figure 4:**
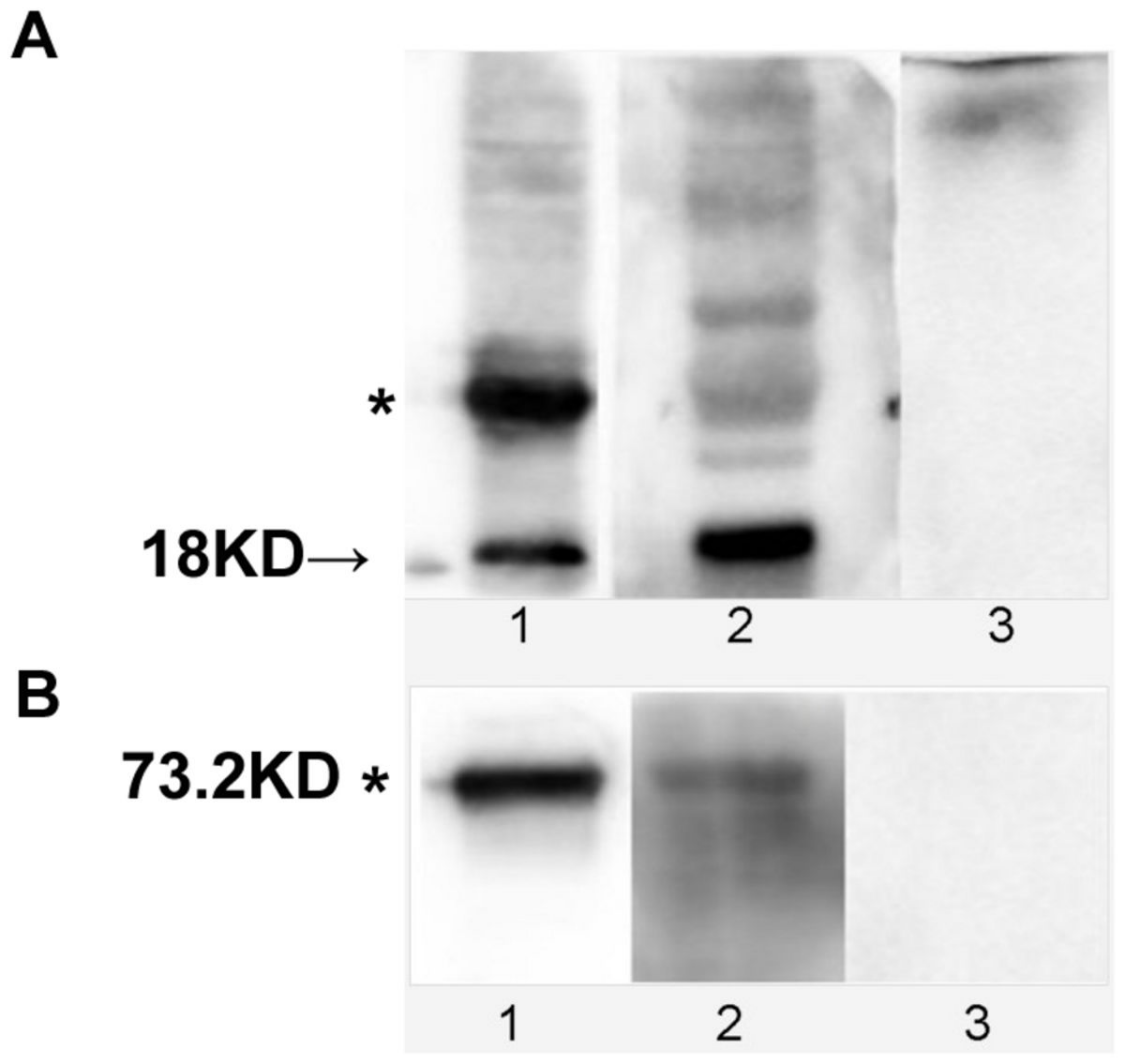
Immunoblot and Far-Western immunoblot analysis demonstrate that Eppin and Fibronectin can combine with each other. A. Immunoblot analysis demonstrating that Eppin binds Fibronectin. Lanes 1: protein of 18-kDa recombinant Eppin (arrow) and multimer forms (*) and were recognized by the rabbit Eppin antibody. Lanes 2: rEppin blot incubated in rFn overnight, washed, and probed with anti-Fn mAbs; lane 3: rEppin blot incubated without rFn overnight, washed, and probed with anti-Fn mAbs. The position of molecular weight standards (kDa) is indicated on the left. B. Far-Western immunoblot analysis demonstrating that rFn on a blot will bind Eppin. Lane 1: protein of 73.2-kDa recombinant rFn (*) blot probed with anti-Fn mAbs; lane 2: rFn blot incubated in r-Eppin, washed, and probed with rabbit Eppin antibody; lane3: rFn blot probed with rabbit Eppin antibody.

 Eppin-Fn binding can be demonstrated by far-Western blotting. Figure 4A (lane 1) demonstrates the monomer (arrow) and multimer forms (*) of rEppin recognized by the rabbit Eppin antibody. When the blot is incubated in rFn, washed, and probed with anti-Fn mAbs, all forms of rEppin bind rFn Figure 4A (lane 2). Anti-Fn mAbs does not recognize rEppin Figure 4A (lane 3). Similarly, rFn on a blot binds rEppin by far-Western blot analysis (Figure 4B). The 73.2-kDa rFn are recognized by anti-Fn mAbs Figure 4B (lane 1). When the blot is incubated in rEppin, washed, and probed with Anti-Eppin, the rFn binds rEppin Figure 4B (lane 2). Anti-Eppin does not recognize rFn Figure 4B (lane 3).

### Colocalize of Eppin and Fn on human ejaculate spermatozoa using immunofluorescence

**Figure 5:**
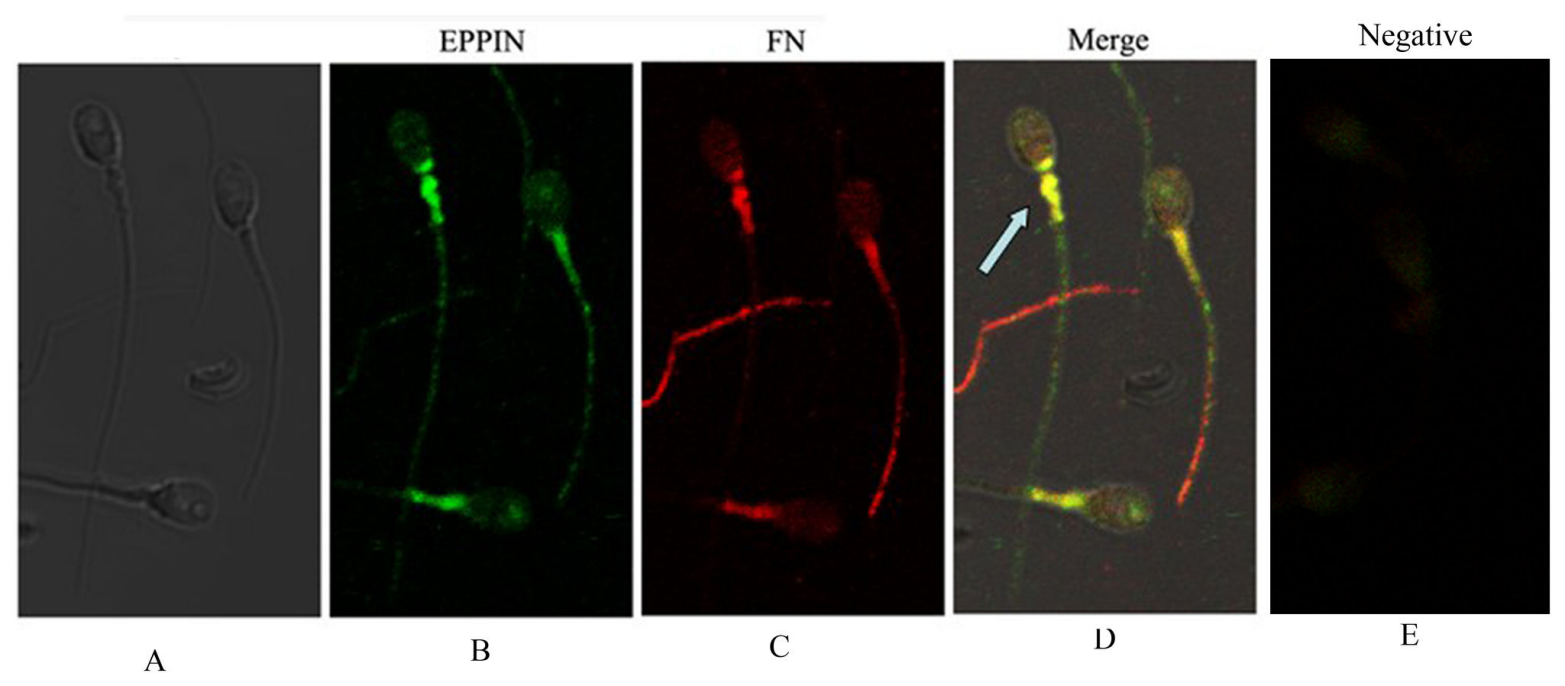
Immunolocalization of Fn and eppin in the sperm. A Phase contrast image of spermatozoon. B. Immunolocalization of Eppin on the head and tail of ejaculate human spermatozoa with anti-Eppin antibodies and detected with fluorescein isothiocyanate (FITC)-conjugated goat anti-rabbit antibodies (green). C. Immunolocalization of Fn on the head and tail of ejaculate human spermatozoa with anti-Fn antibodies and detected with Cy3 (cyanine-3)-conjugated goat anti-mouse antibodies (red). D. Dual-immunofluorescence staining of Fn (red) and Eppin (green) in human sperm showing that the strong colocalization (→) is seen in the postacrosomal and midpiece region of the head (yellow areas). E. Control exposure with fluorescein isothiocyanate (FITC) conjugated goat anti-rabbit IgG and Cy3 (cyanine-3)-conjugated anti-mouse IgG, with isotype mouse antibody and rabbit antibody instead of primary antibody.

 To determine whether the Eppin-Fn complexes was present on ejaculate spermatozoa, washed ejaculate spermatozoa were double labeled with either anti-Eppin and anti-Fn. As shown in Figure 5(A-E), previous research had suggested that Eppin and Fn were present on ejaculate spermatozoa [4,7]. As shown in Figure 5(B.C), immunolocalization of Eppin (green) and Fn (red) on the tail of ejaculate human spermatozoa. On individual spermatozoa, Eppin-Fn complexes are often seen to strongly colocalize (arrow, yellow) on the tail and particularly in midpiece region (yellow, Figure 5D). Control exposure with fluorescein isothiocyanate (FITC) conjugated goat anti-rabbit IgG only, without primary antibody (Figure 5E).

### Co-immunoprecipitation-western blot analysis of extraction from human sperm proteins

**Figure 6:**
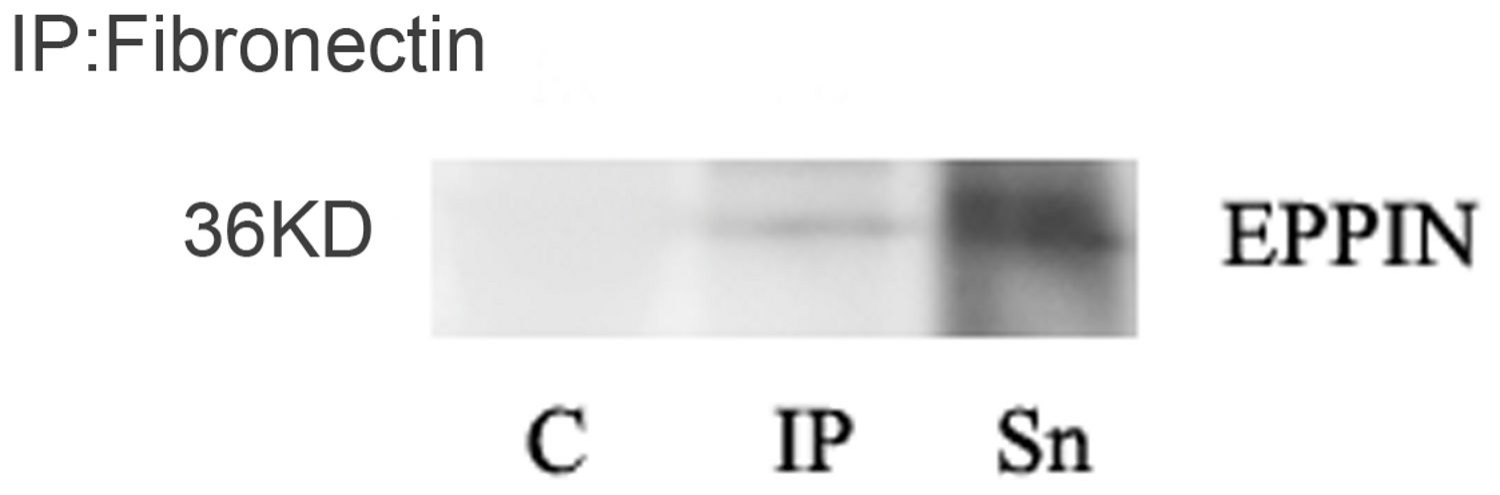
Fn and Eppin interact in the spermatozoa. Anti-Fn mAbs antibody was linked to Plus-agarose beads followed by incubation with 500 µg spermatozoa lysate. The antibody used for immunoprecipitation indicated that Eppin in the spermatozoa lysates was complexed with Fn. Unbound (Sn), or bound (IP) proteins were separated by PAGE. C indicates proteins bound to Plus-agarose linked to normal rabbit IgG as a negative control.

 In order to determine whether these two proteins interact in the spermatozoa, we performed co-immunoprecipitation in spermatozoa lysates. Anti-Fibronectin mAbs were able to co-immunoprecipitate Eppin (Figure 6), indicates that Eppin in the spermatozoa lysates is complexed with Fn (compare Sn, representing unbound protein, to IP, corresponding to protein bound to Fn). The negative control with normal rabbit IgG linked to beads did not precipitate the Eppin proteins.

## Discussion

This study has demonstrated that Eppin is bound to Fn in semen and on human spermatozoa following ejaculation. Four different experimental approaches: 1) MS demonstrates that Fn was the receptor of Eppin, (2) far-Western blotting was used to determine the relation of rEppin and rFibronectin, (3) colocalization was detected on the sperm tail by immunoflorescence, (4) Correalation of Eppin and Fn was proved by co-immunoprecipitation. All demonstrate that Eppin and Fn can bind to each other. 

Our study is the first evidence that Eppin is another receptor of Fn and its binding domain in the N-terminal of Eppin. Immunofluorescence demonstrated that both eppin and Fn were mainly located in the postacrosomal and midpiece region of the head. As reported previously, native Eppin occurs as multimers in both seminal plasma and in the epididymis [1]. These are thought to form by the intermolecular interaction of the 14-cysteine residues. Mass spectroscopy studies on reduced forms of Eppin have determined that the actual mass of the dimer is 33 kDa[4]. The present study demonstrates that, multimer recombinant forms of Eppin can bind rFn (Figure 4), and the native monomers more strongly bind rFn (Figure 4). Moreover, we found that rEppin bond to rFn (amino acids (607-1265), and this sequence contains the only cysteine in human rFn607-1265(Cys-1232), which is necessary for Eppin binding. If a disulfide linkage occurs between them, it might allow several Fn molecules (or fragments) to bind Eppin.

 The physiological significance of the Eppin-Fn complex bound on the surface of ejaculate spermatozoa lies in its ability to provide for the preparation of spermatozoa for fertility in the female reproductive tract, and Eppin may protect spermatozoa from proteolytic attack by allowing cleavage of Sg and Fn bound to Eppin but not of Eppin itself. During human ejaculation, spermatozoa pass through the ampulla of the vas deferens and then move into the proximal extension of the seminal vesicle and finally enter into ejaculatory duct. At this juncture spermatozoa are first mixed with copious secretion from the seminal vesicles. Thereafter the spermatozoa and seminal fluid is mixed with prostatic secretions when they enter into the prostatic urethra. It can be imagined that after spermatozoa enter into the ejaculatory ducts their surface Eppin would be saturated by binding with Sg and Fn[22]. This process inhibits human sperm capacitation, making the initial ejaculated spermatozoa be in an immotile state. Purified plasma Fn, added at various concentrations to a preparation of live spermatozoa, was found to inhibit sperm motility in a dose-dependent manner[23,24]. During semen liquefaction, physiologically PSA hydrolyzes Sg and Fn to increase sperm motility[25,26]. Male monkeys immunized with recombinant Eppin were infertile[6]. Monkey semen appeared to be unable to be liquefied and the sperm motility significantly decreased. It has been shown that Anti-Eppin antibodies disrupt the Eppin-Sg complex and inhibit the proper removal of Sg by PSA[27]. Thus, we conclude from our studies that Anti-Eppin antibodies may also disrupt the Eppin-Fn complex to influence the semen coagulation and liquefaction. 

 In conclusion, the Eppin-Fn complex found on human ejaculate spermatozoa is the part of a larger network of protein complexes on the sperm surface that provides a protective shield before capacitation in the female reproductive tract. Such sperm-coating proteins function in inhibition of proteases that may directly attack the sperm plasma membrane. Many literatures have showed that Fn had an essential role in spermatozoa capacitation and sperm-oolemmal adhesion[7,28,29]. Here our study demonstrated that Fn may involve in semen coagulation and liquefaction and by binding to sperm surface eppin. The detailed molecular mechanism needs to be further investigated. 
